# Comment on Bunmaprasert et al. Reducible Nonunited Type II Odontoid Fracture with Atlantoaxial Instability: Outcomes of Two Different Fixation Techniques. *Int. J. Environ. Res. Public Health* 2021, *18*, 7990

**DOI:** 10.3390/ijerph19095018

**Published:** 2022-04-20

**Authors:** Ayhan Kanat

**Affiliations:** Department of Neurosurgery, Medical Faculty, Recep Tayyip Erdogan University, Rize 53100, Turkey; ayhankanat@yahoo.com; Tel.: +90-5065855139; Fax: +90-464-2130497

I read with great interest the paper of Bunmaprasert et al. [1]. They operated on 21 patients with atlantoaxial instability and compared the outcomes of two different fixation techniques. In the mentioned study, the neurological status of patients after the procedures was not evaluated. I think that this is an important issue. I congratulate the authors, because they achieved a good result, and the bony fusion rate was 100% in both groups. The postoperative radiological view may have been good, but this does not always show that everything is going well. The patient’s clinical condition, such as postoperative chronic pain, disability, and ability to return to his or her previous job is also important [2].

As the authors stated in the limitations section, the study size was relatively small to provide a precise estimation of the confidence intervals of the fusion rate and fusion time, and might not have been enough to conclude the absence of serious surgical endpoints. Despite a small sample size of the study, I believe that this kind of study has importance. In studies with many patients and many centers or neurosurgeons, pooling data from many neurosurgeons or centers resolves the problem of insufficient patient numbers, but using patients from many neurosurgeons may make it harder to ensure a stable condition [3]. 

Normal C1-2 articulations are responsible for approximately 50% of cervical spine rotation [4]. Therefore, stabilization of C1-2 articulations is an important issue [4]. Atlantoaxial fusion procedures for atlantoaxial instability are sometimes are not easy to perform in some situations, such as when there are vertebral artery anomalies or in the presence of ponticulus posticus or arcuate foramen anomaly. Recently, a study has been published about the effect of ponticulus posticus anomaly on the occurrence of odontoid fractures of the C2 vertebra [5].

While screwing this articulation, there is also a potential risk of injury to the vertebral artery. In one of the largest series about posterior C1–C2 transarticular screw fixation, bilateral C1–C2 screws could not be placed in 16 patients (13.2%) out of 121 patients because of an anomalous vertebral artery (n = 13) (10.7%) or other pathological abnormalities [6]. I wonder whether these authors were met with such an anomaly in the 21 operated patients.
